# Brief floodplain inundation provides growth and survival benefits to a young-of-year fish in an intermittent river threatened by water development

**DOI:** 10.1038/s41598-023-45000-x

**Published:** 2023-10-18

**Authors:** Oliver P. Pratt, Leah S. Beesley, Bradley J. Pusey, Daniel C. Gwinn, Chris S. Keogh, Michael M. Douglas

**Affiliations:** 1 School of Agriculture and Environment, The University of Western Australia, Perth, 6009 Australia; 2 Biometric Research, South Fremantle, 6162 Australia

**Keywords:** Freshwater ecology, Wetlands ecology

## Abstract

Riverine floodplains are highly productive habitats that often act as nurseries for fish but are threatened by flow regulation. The Fitzroy River in northern Australia is facing development, but uncertainty exists regarding the extent to which floodplain habitats deliver benefits to fish, particularly given the brevity of seasonal floodplain inundation. We investigated the growth rate of young-of-year bony bream (*Nematalosa erebi*) in main channel and ephemeral floodplain habitats using age derived from otolith daily increments. We also investigated potential mechanisms influencing growth and modelled the consequences of differential growth rate on survival. Our results revealed higher growth occurred exclusively on the floodplain and that zooplankton biomass was the best predictor of growth rate. Modelling indicated that elevated growth rate in high-growth floodplain pools (top 25th percentile) could translate into substantial increases in survivorship. The positive effect of zooplankton biomass on growth was moderated under highly turbid conditions. Temperature had a minor influence on growth, and only in floodplain habitats. Our results indicate ephemeral floodplain habitats can deliver substantial growth and survival benefits to young-of-year fish even when floodplain inundation is brief. This study highlights the need to ensure that water policy safeguards floodplain habitats due to their important ecological role.

## Introduction

Globally, rivers and their biota are threatened by water extraction and flow regulation^1,2^. In lowland river reaches, flow regulation typically reduces the movement of water from the main channel onto the floodplain^3^, reducing the availability and persistence of floodplain habitats and adversely impacting riverine energetics and biota^4^. Fish often use floodplain habitats as nurseries because the slow-flowing waters, abundant food (typically zooplankton) and low densities of fish predators increase larval survival^5^. Moreover, warm water temperatures on the floodplain enhance survivorship as they encourage rapid growth^6^, enabling individuals to pass quickly through the larval and juvenile life phase where mortality is strongly size dependent^7^.

In the rivers of dryland and tropical Australia, floodplain inundation is important for fish production^8–10^. However, research has focussed primarily on floodplain systems that hold water for long periods and none have quantified the benefits of the floodplain on fish growth or survival. Benefits are likely to differ among systems and species depending on the duration and extent of inundation, the magnitude and predictability of flooding, and individual species requirements^11,12^. Extended floodplain inundation brings fish into contact with a greater variety and abundance of food resources, providing opportunities for improved growth^13^. Indeed, zooplankton are often in high abundance in floodplain habitats compared to the main river channel^14^ and provide a calorie dense food resource for many fish species in northern Australia^15^. Brief inundation could limit floodplain benefits for fish because the time available for benefits to be realised is restricted compared to systems where floodplain inundation is prolonged. Floodplain benefits are also likely to vary through time, with benefits materialising in the wake of flood events and diminishing as habitats shrink and competition for resources intensify^16,17^. For this reason, many species use flooding as a cue to spawn, thereby ensuring that larval hatching coincides with optimal conditions for growth^12,18^. The Fitzroy River, located in the Kimberley region of northern Western Australia, experiences brief floodplain inundation (days to weeks) compared to other large rivers in the Australian wet-dry tropics (e.g. the East Alligator River, up to 6 months; the Daly River, up to 4 months, and; the Mitchell river, up to 2 months^19,20^) and the tropical/ sub-tropical rivers of South America^21^, where water may remain on the floodplain for several months. Deep water creek systems on or adjacent to the floodplain (but separate from the main channel) hold water year round^10^, however it is the numerous ephemeral floodplain pools in topographic depressions and minor creek systems that are least well-studied. Many of these pools will dry completely resulting in extirpation of aquatic organisms, while others will persist until hydrological connectivity is restored the following wet season, allowing fish to return to the main channel or seek alternative habitat on the floodplain. The persistence of ephemeral floodplain pools is largely dependent on the timing, magnitude and spatial extent of wet season hydrology (O. P. Pratt pers. comm.). Despite the brief nature of flooding in the Fitzroy River, it is still possible that fish that undergo early life stages in ephemeral floodplain pools will receive growth and survival benefits, particularly if they hatch soon after flooding occurs.

The clupeid bony bream (*Nematalosa erebi*) (Günther)*,* is a habitat generalist and Australia’s most widely distributed freshwater fish^22^. It is found in high abundance, makes up a significant proportion of fish biomass and is often present in the most marginal of ephemeral floodplain habitats^23^. Bony bream play an important role in riverine food webs as they are a key prey item for both aquatic and terrestrial predators^24^. They are also one of the few species that (as adults) consume detritus (leaf litter) thereby facilitating the transfer of terrestrial production into the aquatic food web^22^. As juveniles, bony bream predominantly feed on zooplankton^15,22,24^. Although flooding may stimulate bony bream to spawn, they can spawn at multiple times throughout the year, including during the falling limb of flood events, at times of low flow and during periods of prolonged drying^25–27^. Spawning occurs in both riverine and floodplain habitats and so they provide an ideal model to investigate the energetic benefits of the floodplain compared to the main channel. They also provide an opportunity to assess whether benefits on the floodplain vary between fish born early or late in the flood cycle.

Little is known about the extent to which fish in Northern Australian rivers with short floodplain inundation, such as the Fitzroy River, gain energetic benefits by occupying floodplain habitats. The present study investigates whether the floodplain provides a growth rate benefit to young-of-year bony bream. We hypothesise that: (1) young-of-year fish in ephemeral floodplain pools will grow faster than those in the main channel; and that this will translate into higher survival in floodplain habitats. We expect that (2) high zooplankton abundance and warmer water temperatures in floodplain habitats will be the primary mechanisms driving the pattern. Finally, we expect that (3) there will be an energetic benefit afforded to fish that hatch and undergo early life stages during flood events compared to those that hatch after flooding has finished. Increased understanding of the benefits associated with floodplain habitats will assist the creation of water policy that protects flows and habitats important to healthy river functioning.

## Methods

### Use of animals

This research was carried out under Fisheries exemption 191-2009-27 (FARWH) and 2974 (NESP), Animal Ethics permits RA/3/100/1536 and RA/3/100/884 (Animal Ethics Committee, The University of Western Australia), and the Department of Environment and Conservation permit number SF006973. All field work was undertaken in accordance with the Animal Welfare Act (2002) (Western Australia) and in accordance with ARRIVE (Animal Research: Reporting of In Vivo Experiments) guidelines.

### Study area

**Figure 1:**
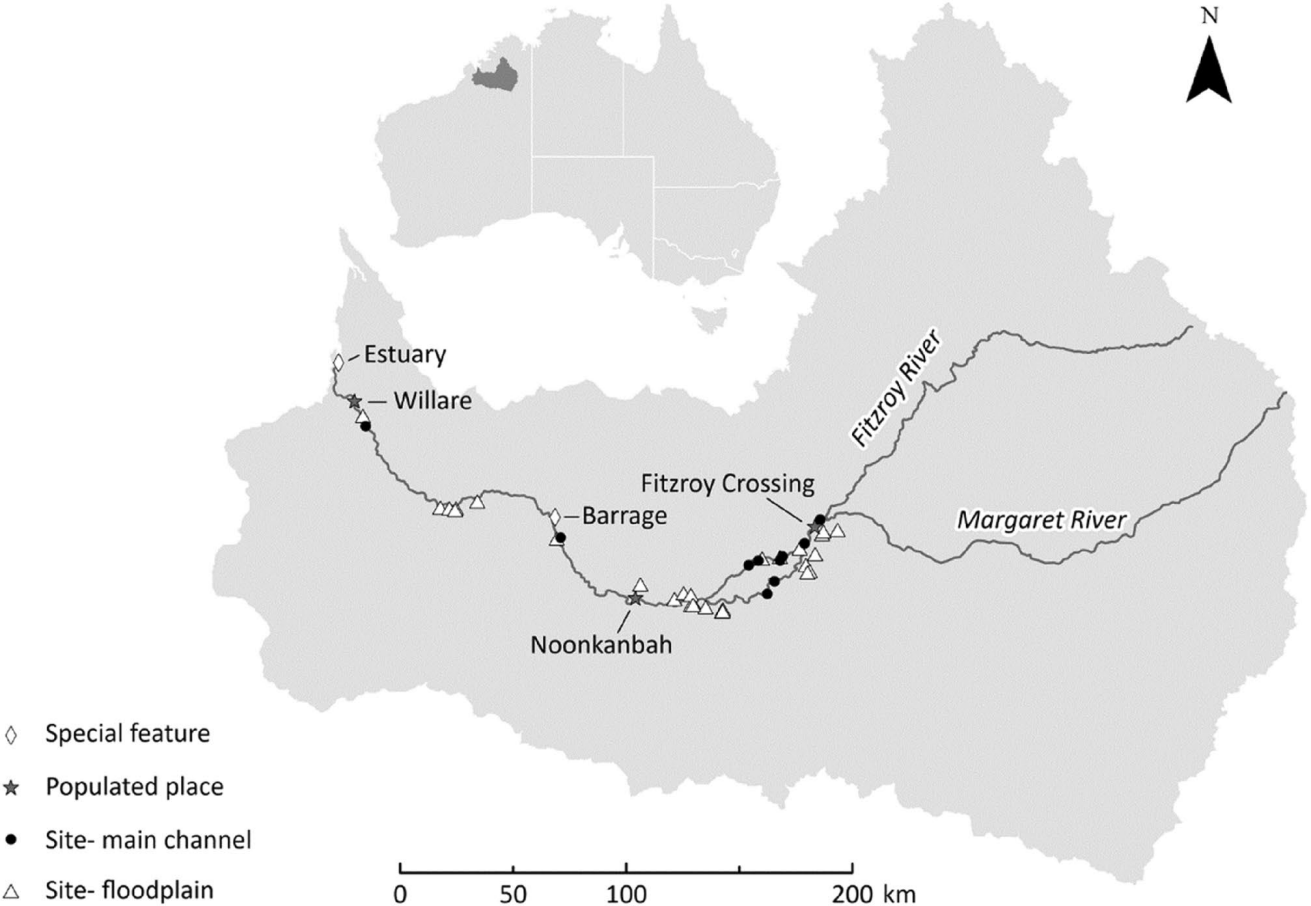
The location of main channel and floodplain sites sampled for bony bream in the Fitzroy River, Kimberley, Western Australia.

The Fitzroy River is located in the wet-dry tropical region of the Kimberley, Western Australia. The river is ~ 700 km in length and situated within a 94,000 km^2^ catchment. The lowland section of the river is characterised by a meandering channel with the occasional large anabranch. The river experiences distinct wet and dry seasons, with a mean annual discharge of 6600 GL^28^. Flow varies markedly among years and its hydrology is classified as wet season highly intermittent by Kennard, et al.^29^. Wet season flows occur between December and April, connecting pools in the main channel and delivering water into floodplain distributary creeks. Overbank flooding which inundates the floodplain is relatively brief (i.e., days to weeks) compared to other rivers in northern Australia^19^. During the dry season, from May to November, the main channel disaggregates into a series of clear water pools that are connected by long shallow runs. Pools on the floodplain also contract with many of them becoming increasingly turbid as the dry season progresses. Flow in the river is relatively unmodified with the only regulatory structure being the 3 m-high Camballin barrage (an instream weir that diverts water into nearly Uralla Creek for irrigated agriculture), situated approximately midway along the lower river (Fig. 1). The river has been identified as having potential for water resource development to support irrigated agriculture^30^. If development goes ahead, water from distributary creeks may be diverted into off-stream storages. This process may decrease hydrological connectivity between the river and floodplain, reducing the area, depth and duration of seasonal floodplain inundation^31^.

### Study design and sampling methods

**Figure 2:**
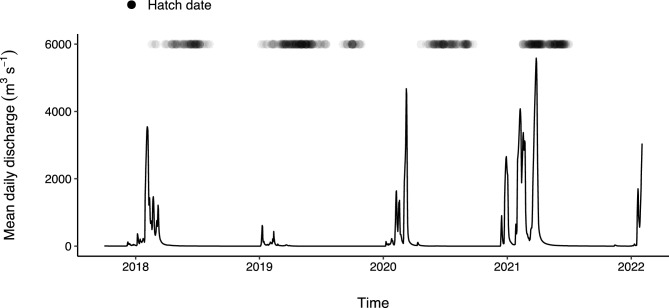
Mean daily discharge from Willare gauging station (Station Number 802008, Australian Bureau of Meteorology) during study period. Black dots represent hatch date of fish included in analysis. Darker shading signifies more individuals hatched.

Young-of-year bony bream < 100 mm standard length were collected during the dry season (June-November) over a four-year period (2018–2021) that exhibited considerable variation in wet season flow. For instance, flooding occurred as a single pulse in years 2018 and 2019, as a double pulse in 2020 and as three separate pulses in 2021 (Fig. 2) (mean daily discharge data from Willare Gauging Station No. 802008, Australian Bureau of Meteorology). Fish were collected from a total of 37 sites: 10 in the main channel and 27 from ephemeral floodplain pools. Sites spanned the lower 350 km of the river, between Fitzroy Crossing and Willare (Fig. 1). A subset of sites were surveyed over multiple years, making a total of 53 site*time sampling events (Table 1). Fish were collected using seine nets (mesh size 7 or 9 mm) and/or cast nets (mesh size 15 mm) and up to 10 individuals spanning the size range present at each site were euthanased by submersion in a solution with AQUI-S™, frozen and transported to the laboratory for processing.

**Table 1:** Fish sampled from each habitat over the four-year sampling period. Values in parentheses represents the number of sites from which fish were sampled.

Habitat	2018	2019	2020	2021
Floodplain	43 (7)	70 (7)	85 (10)	113 (14)
Main Channel	24 (3)	75 (8)	0	13 (4)

Environmental parameters known to influence fish growth were measured at each site prior to sampling. Turbidity was measured at 15 cm depth using a YSI ProDSS (Xylem, U.S.A) at three locations within each site and the mean value used in statistical analysis. Zooplankton were sampled prior to fishing but were only collected during the latter two years (2020–21), i.e., at 4 main channel and 24 floodplain sites. We used a plankton tow net with a 100 µm mesh to target large zooplankton which are known to be an important food resource for juvenile fish^32^. Three replicate open water samples, each 40 L in size, were poured through the net. Samples were spread across the site. Seston in the net was washed down the side of the net and into a 50 ml vial and preserved in 70% ethanol. Mean water depth at each site was estimated from a series of point measurements (13–45 per site) along transects (2–5 per site).

Additional environmental parameters relating to water temperature and flood events were collected remotely. To describe the thermal conditions that fish were exposed to, we used the degree-days method^33^. We considered this more robust than spot measurements of temperature for several reasons. Firstly, as temperature displays considerable diurnal variation a single spot measurement would include this noise. Secondly, a spot measurement only describes current conditions and doesn’t represent the historical conditions that the fish has been exposed to, whereas the degree-days method derives an index of the metabolically relevant thermal energy that an individual has experienced over time, relative to a base temperature (T_0_)^34^. A strong linear relationship is known to exist between degree-days and juvenile fish growth^35^. As direct in-situ measurements of water temperature were not available for the sites sampled, air temperature was used as a surrogate, as per Honsey, et al.^34^. Given the majority of sites were shallow floodplain pools (mean depth 0.5 m) with minimal shading, it is likely that air and water temperature were strongly coupled. Daily minimum and maximum ambient air temperature data (°C) were downloaded from the Australian Bureau of Meteorology for the weather station at Fitzroy Crossing (Site Number 003006) for the study period. The cumulative air degree-days (hereafter degree days) experienced for each fish over its lifetime was calculated based on date of collection and hatch date derived from otolith daily increment analysis. Chezik, et al.^33^ showed that a broad range of T_0_ values can effectively explain variation in juvenile fish growth and suggested that precise T_0_ values are unwarranted. We used a T_0_ value of 30 °C after assessing a range of values (15, 20, 25, 30, and 35 °C) within the water temperature range in which bony bream are found (12–38 °C ). Linear regression analysis was used to identify which T_0_ best explained growth rate data, however our results showed little sensitivity to changes in T_0_. To investigate whether fish that hatched during a flood event gained an energetic advantage over those that hatched post flooding, we collected data describing the number of wet season days each individual experienced during its life. The wet season was defined as the period of time when floodplain inundation occurred which was determined from visual inspection of daily satellite images (spectrum RBG NIR, resolution 3 m)^36^ in the area surrounding each site. The number of wet season days was calculated from the temporal overlap between periods of floodplain inundation and the lifespan of each individual fish.

### Laboratory procedures: aging

Growth rate data requires information on fish size-at-age. Fish age was determined by counting daily increments in otoliths, the calcareous structures found in the inner ear of fish. Validation of otolith increment counts as a measure of age is commonly required for growth studies of young-of-year fish. While this validation has not been conducted for bony bream, numerous studies in both marine and freshwater environments have consistently shown daily accrual of otolith increments^37–39^, including in species within the same sub family as bony bream^40^. Furthermore, the increment count—body length relationship observed in our dataset align well with those of individuals from the opposite end of the species range (Macquarie River, Murray-Darling Basin) published by Stocks, et al.^41^.

Standard length (mm) was measured and recorded for each fish (n = 423) under laboratory conditions within a month of being frozen. Sagittae otoliths were removed in the laboratory and dried and stored in Eppendorf containers. Otolith preparation for daily age estimation followed Robbins and Choat^42^. The proximal and distal surfaces of each otolith were ground down using 1200-grit lapping film to expose daily growth increments. Otoliths were viewed under 400× magnification using a Leica DM-3000 microscope. Age was estimated for each individual by counting daily increments from a hatch mark (~ 15 µm from the primordium) to the outer edge of the otolith section. Each otolith was aged twice, if the two ages were inconsistent a final ‘agreed’ age was determined with a third read. A sub-sample of n = 126 otoliths was aged a final time to quantify intra-reader error, expressed as the average percentage error (APE). The derived APE was 2.1%, well below the upper limit of 3% considered to indicate acceptable precision^43^.

### Laboratory procedures: estimating zooplankton biomass

Zooplankton field samples were stained with rose bengal, filtered through a 100 µm sieve, rinsed, and transferred into a container where deionised water was added to a volume of 50 ml. Samples were agitated to lift zooplankton into suspension and to homogenise the sample. Five 1 ml aliquots were taken from each sample and transferred into a Bogorov chamber for analysis under a Leica S9i stereo microscope. Zooplankton samples consisted almost entirely of Copepoda (~ 87% of total number counted), Cladocera (~ 12%) and Ostracoda (< 1%). Each zooplankter was counted and measured using the ICC50 W camera kit and Leica LAS V4.12 software. Copepoda were measured from the head to the base of the caudal setae (including rami in the case of adults), Cladocera from the head to the base of the body excluding the tail spine, and Ostracoda were measured along the long axis of the carapace. Dry mass (µg) for each individual was calculated from the ‘pooled’ length–weight regression equations from Bottrell, et al.^44^ (Copepoda and Cladocera) and Shmeleva^45^ (Ostracoda). Zooplankton dry mass for each site was determined from the sum of the dry mass of all individuals in each 5 mL aliquot from all three site replicates. This value was converted to dry mass per volume i.e. µg L^−1^ for statistical analysis. We use dry mass as a surrogate of biomass instead of abundance as it is more relevant to energetics and growth^44^.

### Statistical approach

We explained the variation in growth rate of young-of-year fish with a linear mixed-effects model. The dependent variable was growth rate, calculated as SL/age, which assumes linear growth common for juvenile fish^46^. Covariates were included as independent variables to assess the influence of food, temperature, habitat and flooding on growth rate. These included main effects for habitat (floodplain or main channel), zooplankton biomass (dry mass (µg L^−1^)), degree days, and the number of ‘wet season days’ each individual experienced. We included an interaction between zooplankton biomass and turbidity to account for the possible moderating effects of turbidity on the hunting success of visual predators^47^. Turbidity was represented as a binary variable that discriminated between values > or < 400 NTU, which approximates the level of turbidity when substantial changes in predation, behaviour, and condition tend to occur for a variety of fishes^48–50^. A degree day-habitat interaction term was included to account for the possibility that temperature differences could manifest differently in main channel and floodplain pools. For instance, we expected that shallow floodplain pools which receive minimal shading from riparian vegetation would be warmer than deep main channel pools that receive greater shading^51^. Zooplankton data were not available for all sites where bony bream were sampled. To account for these missing data, we set their values to zero and estimated a separate residual error for sites with and without zooplankton data. This allowed us to include the full dataset in the analysis without inducing bias in the parameter estimates of the model. Both ‘site’ and ‘year’ were included as random effects to account for the non-independence of fish within the same site and possible non-independence of growth rate among years. All continuous covariates were centred on zero and scaled to one standard deviation. Correlation between covariates was < 0.7 (Pearson correlation coefficients). We transformed the dependent variable by adding 10 and taking the natural logarithm. This transformation allowed us to meet the assumptions of homoscedasticity without changing the shape of the relationship between dependant and independent variables.

The growth rate model was fitted in a Bayesian framework using the Gibbs sampler JAGS version 4.3.0^52^ called within program R version 4.1.2^53^ with package R2jags version 0.7–1^54^. Two MCMC chains were run for 100,000 iterations with a thin rate of 10. The first 50,000 iterations were discarded leaving a posterior sample of 10,000 for inference. We considered chains converged when the $\widehat{R}$ < 1.1^55^. Model fit was diagnosed with visual inspection of residual and Q-Q plots (see supplementary material). Standard deviations of random effects were given Student t-distribution priors with $\sigma =1.57$ and $\nu =7.763$ as per Gelman and Hill^56^. To protect from model overfitting we performed model regularisation with Stochastic Search Variable Selection (SSVS)^57,58^. This process invokes parameter shrinkage on effect parameters with conditional priors specified as, $\theta |w\sim \mathrm{Normal}\left(0,{\sigma }^{2}|w\right)$, where $\theta$ is the covariate effect parameter and $w$ an inclusion parameter that can take the value of 0 or 1. When the inclusion parameter $w=1$ the variance of the prior ${\sigma }^{2}=100$, which specifies a standard normal uninformative prior for $\theta$. When $w=0$, the variance ${\sigma }^{2}=0.01$ which generates a region of high probability at $\theta =0$, effectively excluding the parameter from the model. We specified a Bernoulli prior for $w$ with probability 0.5 that assigns equal probability of inclusion or exclusion of each covariate of our model. The posterior mean of $w$ can be interpreted as support for a non-zero parameter value where values > 0.5 indicate strong support for inclusion^59^. A further advantage of this method is that model predictions and effect estimates are automatically model averaged, optimizing the predictive properties of the model^57,58^. We considered covariate effects statistically different than zero when 95% Bayesian credible intervals of the model averaged posterior samples did not include zero (approximating $\alpha \le 0.05$).

To aid in the interpretation of any differences in growth rate between habitats, we assessed differences in zooplankton biomass with a non-parametric bootstrap process. We generated bootstrap samples of zooplankton dry mass data (µg L^−1^) collected from floodplain and main channel sites by resampling with replacement for 10,000 iterations. We considered zooplankton biomass statistically different between habitats when 83% bootstrap confidence intervals did not overlap (approximating $\alpha \le 0.05$).

### Survival consequences

As survival in fish is closely linked to size and growth^60^, we contextualise our results by deriving the expected survival consequences of variation in growth rate between habitats. This was achieved by first predicting average total length (${L}_{t}$) at age up to age of maturation (550 days old) for each habitat, using a linear model,

1 $${L}_{t}=mx+b$$

where $m$ is average growth rate (i.e. the slope of the relationship), $x$ is age in days and $b$ is the size at hatching (3 mm^25^ i.e. the intercept). Mortality rate at age (${M}_{t}$) in each habitat was then predicted per Lorenzen^60^ as,

2 $${M}_{t}={M}_{r}\left(\frac{{L}_{r}}{{L}_{t}}\right)$$

where ${M}_{r}$ is instantaneous mortality at reference length ${L}_{r}$. We approximated ${M}_{r}$ using R package FishLife^61^. Survivorship (${S}_{t}$, proportion surviving to age *t*) was calculated recursively as ${S}_{t}={S}_{t-1}{e}^{-{M}_{t-1}}$.

## Results

**Figure 3:**
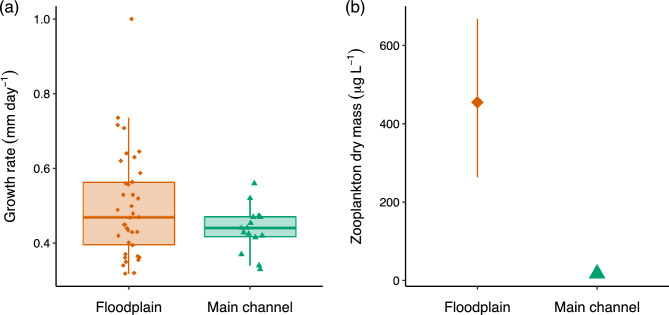
(**a**) predicted growth rate of bony bream in floodplain (diamonds) and main channel (triangles) habitats. Box limits span the interquartile range with the median represented with a coarse horizontal line. (**b**) zooplankton dry mass in floodplain (diamonds) and main channel (triangles) habitats. Symbols represent estimates of parameter means and their associated 95% confidence intervals derived from non-parametric bootstrap with replacement of zooplankton data. Error bars (CI 3.09, 9.28) in main channel habitat are too small to visualise in (**b**).

Thirty-eight sampling events occurred in floodplain pools (average depth 0.50 m) and 15 in main channel pools (average depth 1.35 m). A total of 423 bony bream were collected, with an average length of 46 mm (SL) and an average age of 102 days (size range 15–97 mm, age range 24–234 days). A total of 12,236 individual zooplankton were counted, measured and identified. The range of zooplankton biomass was large in floodplain sites, between 0.62 and 2967 µg L^−1^, compared to main channel sites where zooplankton biomass varied little, between 3 and 9 µg L^−1^. Mean zooplankton biomass was 70 times greater in floodplain pools (454 µg L^−1^) than in the main channel (6 µg L^−1^) (Fig. 3b). This difference was statistically significant (p < 0.001, CI 253.21, 660.73).

### Growth rate analysis

**Figure 4:**
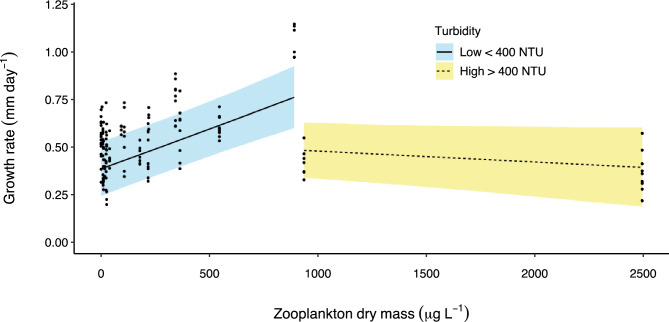
Model predicted bony bream growth rate vs zooplankton biomass in floodplain pools under different turbidity scenarios. This figure is a graphical representation of the zooplankton biomass*turbidity interaction effect in the growth model. There was no overlap in zooplankton dry mass between low (< 400 NTU) and high (> 400 NTU) turbidity sites. Area of shading surrounding each line represents 95% Bayesian credible intervals.

**Figure 5:**
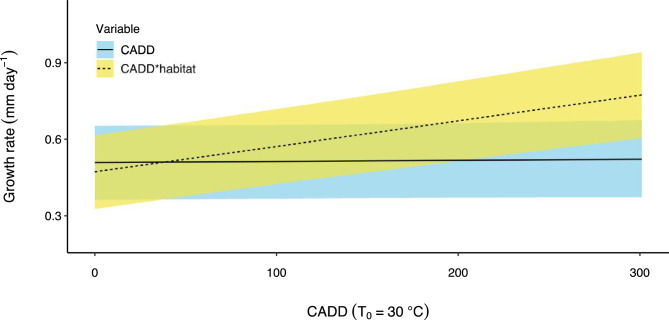
Non-significant relationship between growth rate and cumulative air degree days (CADD) (solid line) and significant relationship between growth rate and CADD*habitat interaction (dashed line). The interaction term shows the effect of the floodplain on CADD. Area of shading surrounding each line represents 95% Bayesian credible intervals.

On average, fish in floodplain habitats grew at the same rate as those in the main channel as indicated by 95% Bayesian credible intervals that overlap zero for the mean effect of ‘habitat’ (Table 2). However, the growth rate of fish on the floodplain was more variable and a quarter of all fish on the floodplain (upper 25th percentile) grew faster than fish in the main channel (Fig. 3a). The maximum growth rate obtained by a young-of-year fish was 1.8 times greater on the floodplain compared to the main channel (1 vs. 0.56 mm day^−1^ respectively) (Fig. 3a). Indeed, ranked order of predicted growth rate shows only one main channel site in the top 15 of 53 site*time sampling combinations. Several mechanisms contributed to increased growth in some floodplain habitats, but the primary influence was food availability. For instance, predicted growth rate increased by 0.04 mm day^−1^ for every 100 µg L^−1^ increase in zooplankton biomass (Fig. 4). In floodplain pools where zooplankton biomass was low, growth rate was slow (Fig. 4) and similar to that observed in the main channel where maximum zooplankton biomass was only 9 µg L^−1^. The effect of zooplankton biomass on growth rate was significant as evidenced by a parameter inclusion probability of 1 and 95% Bayesian credible intervals that exclude zero (Table 2). However, the positive effect of zooplankton biomass on growth rate was moderated in highly turbid conditions. For instance, in pools where turbidity was > 400 NTU, predicted growth rate was below 0.5 mm day^−1^ despite high zooplankton biomass (dashed line, Fig. 4). This moderating effect is significant as evidenced by parameter estimates of the zooplankton biomass*turbidity interaction term (Table 2). The effect of temperature on growth was relatively minor and was complex. Temperature was only related to growth in floodplain habitats (Fig. 5). For instance, the main effect ‘degree days’ had a low parameter inclusion probability and an effect size of 0, whereas the interaction term ‘degree days*habitat’ had a high parameter inclusion probability (1) and credible intervals that didn’t overlap zero (Table 2). The positive effect size of the interaction term indicates that in floodplain habitats, the more thermal energy experienced by an individual, the higher the average growth rate (Fig. 5). We found no evidence that the timing of hatching relative to flooding influenced growth as demonstrated by the negligible effect size of the covariate ‘wet season days’ and its small parameter inclusion probability (Table 2).

**Table 2:** Parameter estimates from bony bream growth rate analysis. SD- standard deviation; LCI- 95% lower credible interval; UCI- 95% upper credible interval; $w$- inclusion probability. $w$ is an indication of the support for the inclusion of a given variable in the best predictive model. ^†^ signifies parameter inclusion in the model that best predicts bony bream growth rate.

Variable	Effect size	SD	LCI	UCI	$w$
Habitat	0.001	0.003	0.000	0.011	0.112
Degree days	0.000	0.001	0.000	0.002	0.147
Zooplankton biomass^†^	0.024	0.004	0.015	0.031	1
Zooplankton biomass*turbidity^†^	−0.027	0.005	−0.037	−0.016	1
Wet season days	0.000	0.000	−0.001	0.000	0.144
Degree days*habitat^†^	0.005	0.001	0.003	0.007	1

### Survival

**Figure 6:**
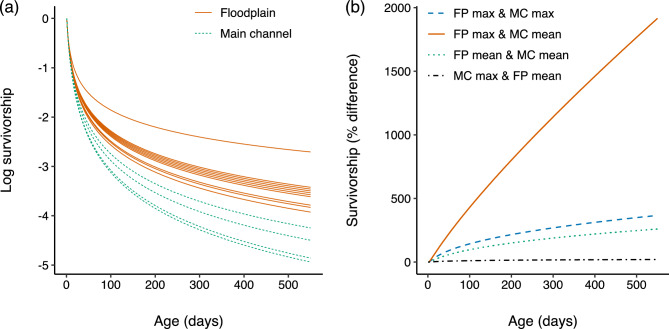
**(a**) log survivorship with age in floodplain (solid lines) and main channel (dashed lines) sites. Sites displayed represent predicted growth rate in high-growth pools (i.e. upper 25th percentile), representing the maximum growth benefit offered in each habitat. (**b)** percent difference in survivorship with age for a combination of floodplain (FP) and main channel (MC) fish growth rates: maximum growth rate FP vs mean growth rate in MC (solid line); maximum growth rate on FP vs maximum growth rate in MC (dashed line); mean growth rate on FP vs mean in MC (dotted line); and maximum growth rate in MC vs mean growth on FP (dot-dash line). Both x-axes extend to 550 days which is the mean age at sexual maturity for bony bream.

Elevated growth for a subset of fish on the floodplain translated into heightened survival, particularly in pools where conditions for growth were optimal (Fig. 6a). For example, survivorship estimates modelled from predicted growth rates in high-growth (i.e. the top 25th percentile) floodplain sites, were consistently higher than survivorship estimates in high growth main channel sites (Fig. 6a). The disparity in survivorship between high growth and low growth pools increased over time (Fig. 6b) because smaller sized fish suffered higher mortality due to their smaller size resulting in a multiplicative effect. At the extreme, a comparison of modelled survivorship from maximum predicted growth on the floodplain with mean predicted growth in the main channel revealed survivorship on the floodplain was 1140% higher after 300 days, the approximate time until floodplain connectivity is restored the following wet season, and 1915% higher after 550 days, the approximate mean age of sexual maturity in bony bream (Fig. 6b). In this scenario, for every one fish that reached sexual maturity in the average main channel site, 20 individuals reached sexual maturity in the optimal floodplain site. While this example represents the maximum floodplain energetic benefit compared to the average main channel benefit, even modest increases in growth can provide meaningful increases in survivorship. For example, the mean growth rate differential between habitats, which was not significant at alpha < 0.05, still resulted in a 258% increase in survivorship at sexual maturity on the floodplain (Fig. 6b), corresponding to a ratio of 1:3 individuals reaching sexual maturity in the average main channel and floodplain site.

## Discussion

This study demonstrates that floodplain habitats in the Fitzroy River have the potential to provide a growth benefit to young-of-year bony bream, an ecologically significant fish species in Australia. High growth occurred exclusively on the floodplain, where predicted maximum growth rate (1 mm day^−1^) was 1.8 times greater than maximum growth predicted for the main channel (0.56 mm day^−1^). The primary mechanism influencing growth rate was greater food availability in floodplain habitats, with average zooplankton biomass more than 70 times greater in floodplain pools. However, in pools where zooplankton biomass was low, bony bream growth rate and modelled survivorship was similar to that observed in the main channel. Modelling revealed that elevated growth in optimal floodplain pools could translate into a 20-fold increase in survivorship to sexual maturity compared to the main channel average. Our findings highlight the important role of floodplain habitats in the growth and survival of young-of-year fish in an intermittent tropical river, even when floodplain inundation is brief. Protecting river-floodplain connectivity and floodplain habitats from the impacts of water resource development should be a critical consideration to ensure the productivity and functioning of the system.

Our finding that young-of-year fish in the Fitzroy River grew faster in certain floodplain habitats than in the main channel, is consistent with studies of juvenile fish growth in other large rivers around the world^6,62^. This is, however, the first time that growth benefits to young-of-year fish on the floodplain have been empirically demonstrated in northern Australia. Growth rates presented in this study are within the same range as those reported for young-of-year bony bream at the opposite end of the species range (Macquarie River, Murray-Darling Basin). Stocks, et al.^41^ reported growth rates of between 0.66 and 1.17 mm day^−1^ based on fork length data and age derived from otolith daily increments, however they did not seek to link growth to environmental factors e.g. habitat, food availability or temperature. Other studies have primarily sought to reveal the benefits of seasonal flooding and river flow for fish growth, recruitment, and diet for a variety of species including bony bream^8–10,19,63^, rather than the direct influence of habitat. The brief nature of floodplain inundation in the Fitzroy River means that ephemeral floodplain pools isolate quickly during the dry season and diverge in their physical and biological characteristics, i.e., depth, turbidity, zooplankton. This habitat diversity, in turn creates a diversity of growth and survival benefits for bony bream. For instance, while fish in an average floodplain pool had similar growth rate to the main channel, those in pools with abundant zooplankton showed considerable growth benefits. Our study predicted that these growth benefits have the potential to translate into large increases in juvenile survival. This assertion is supported by studies elsewhere that have evaluated the factors shaping fish recruitment, where strong recruitment is driven primarily by environmental conditions that influence larval and juvenile survival, such as food availability and temperature^18,64^. Increased survival on the floodplain could mean that fish reared in this habitat make a disproportionally large contribution to the adult spawning stock. It could also promote the transfer of energy from the floodplain to the main channel if hydrological connectivity is restored prior to pool drying. This is consistent with the Riverscape Recruitment Synthesis Model proposed by Humphries, et al.^65^, which suggests that high temperatures and abundant zooplankton on the floodplain favour strong recruitment for periodic and opportunistic life-history strategists, such as bony bream. However, both of these recruitment implications are dependent on growth-related survival benefits being realised, which is not a given. Indeed, it is important to recognise that our survivorship calculations were based solely on size-dependant mortality and did not account for the effects of density-dependant mortality, a process which tends to be strongest in early life stages^66^. Density-dependent mortality can be driven by the depletion of limited food resources i.e. starvation, or can arise due to increased competition for food or habitat availability which promotes risky behaviour and exposure to predators^67^. Our survivorship calculations also did not account for extirpation events in floodplain habitats caused by deoxygenation or pool drying. However, these effects may be balanced somewhat by the increased predation risk that bony bream face in the main channel compared to ephemeral floodplain pools, due to the presence of large predators, such as barramundi (*Lates calcarifer*) and sawfish (*Pristis pristis*) (D. C. Gwinn, work in preparation). Survivorship estimates are also sensitive to values of instantaneous mortality (${M}_{r}$), with potential error increasing with fish age due to multiplicative effects. However, the purpose of our survivorship estimates is to represent the expected average outcome of the habitat growth rate differential to show the potential survival benefits associated with high growth pools only found on the floodplain. We recommend that our survivorship estimates are considered in relative not absolute terms. Ultimately, studies that directly assess survival are needed to confirm the benefits associated with floodplain habitats.

Fish growth can be strongly influenced by the quality and availability of food resources. When food is in limited supply or of low nutritional quality, growth rate can be retarded^68^. In the present study, we provide direct causal evidence that the primary mechanism influencing the growth of young-of-year bony bream in the Fitzroy River is zooplankton biomass. Zooplankton are a highly nutritious food item for juvenile bony bream^15,22,24^ and contribute to the diet of many other fish species in Australia^15^ and elsewhere^69^. The average biomass of zooplankton was 70 times greater in floodplain pools than in the main channel, where zooplankton were largely absent, a finding consistent with other research on rivers in Australia^70^ and globally^71^. Given zooplankton are typically the primary food resource for most larval fishes at the onset of exogenous feeding regardless of adult dietary guild^72^, it is likely that the benefits associated with high zooplankton biomass in floodplain pools are extended to most fish species during early life stages, both in Australia and globally. The low abundance of zooplankton observed in the main channel of the Fitzroy River suggests that zooplankton may originate from egg banks in the sediments of ephemeral floodplain pools^32^ rather than being transported from the main channel by floodwaters. However, not all floodplain pools had high zooplankton biomass. In pools where zooplankton biomass was low, bony bream growth rate was low and similar to that observed in main channel habitats. Our results also revealed that the positive effect of high zooplankton biomass on growth rate was moderated under high turbidity conditions (> 400 NTU). Several studies have demonstrated that reduced water transparency can impact the hunting efficiency of zooplanktivorous fish^47,49^. This may explain slow growth among young-of-year fish in turbid sites despite high zooplankton biomass.

Environmental conditions such as temperature or the timing of flood flows can also influence fish growth^6,65^. In the present study, we found that the more thermal energy an individual experiences over its lifetime the faster its average growth rate. However, this was only observed in floodplain habitats, with deep water and increased shading from riparian vegetation at main channel sites potentially buffering thermal patterns^51^. The importance of temperature on growth was small in this study, this may be due to the indirect way in which it was measured i.e. air temperature. Whilst air temperature and water temperature is likely to be coupled, particularly in shallow floodplain pools with minimal shading, we recommend that future studies install temperature loggers so that in situ data from each site can be used. We found no evidence that time of hatching influenced growth rate. This is in contrast to other studies which have suggested floodplain benefits are best realised soon after flooding but disappear as floodplain habitats shrink and competition for resources increases^16,17^. This finding suggests that initially flooding in the Fitzroy River may be more of a disturbance i.e. a destructive force that displaces organisms and damages habitat^73^. For instance, despite the likely increase in zooplankton biomass associated with terrestrial nutrient input during flooding^74^, it is probable that this food resource will be diluted and obscured by turbid flood flows, reducing foraging efficiency and retarding growth of larval and juvenile bony bream in the period during/ immediately after flooding. Once floodwaters recede, fish and zooplankton are concentrated into isolated, clear-water floodplain pools where hunting efficiency would likely increase and so too fish growth rate.

This study has implications for the management of water resource development in the Fitzroy River and more broadly across northern Australia and further afield. Currently, the Western Australian State Government has a ‘no dam policy’ on the Fitzroy River and its tributaries^75^, therefore floodwater harvesting is the most likely method of surface water extraction. Floodwater harvesting has the potential to decrease hydrological connectivity between the main channel and floodplain^76^, reducing the number and area of floodplain pools^31^. Moreover, the impact of floodwater harvesting will likely be more pronounced in areas where floodplain connectivity is most brief e.g. upstream from Noonkanbah^77^ or where floodplain and main channel aquatic habitat is less abundant such as the lower 100 km of the river^78^. Water resource development in these areas has the potential to negatively impact floodplain pools and therefore habitats where juvenile bony bream and other species thrive^10^. Indeed, historical water development in the main river channel at Camballin barrage has had a negative impact on bony bream populations immediately downstream of the structure^79^. A reduction in floodplain pools and the juvenile bony bream they support will likely translate into reduced food for many important species as bony bream are prey for higher-order consumers including those of recreational and cultural significance e.g. barramundi (*Lates calcarifer*)^79^ and the critically endangered freshwater sawfish (*Pristis pristis*)^24^. They are also a food source for waterbirds, as well as an important baitfish for customary fishing^80^. Furthermore, a reduction in bony bream may have an impact on broader riverine energetics. Mature fish are detritivores, facilitating the movement of terrestrial carbon into the aquatic food web, making them one of few fish species in northern Australia to fill this role^22^. Given the ecological and cultural importance of bony bream across northern Australia and the interest in developing the region’s water resources^30^, it is vital that when water planning policy is being developed, the aspects of the natural flow regime that create and maintain floodplain habitats are protected to safeguard the future of this important habitat. Indeed, this study has implications for the management of riverine systems globally. Given that zooplankton are routinely found in high abundance in floodplain habitats around the world^69,71^, it is likely that the associated growth and survival benefits may apply to other fish species that consume zooplankton^69^, and perhaps to most fish species during the larval life-stage^72^. Maintaining healthy fish populations is a key aim for river managers around the world, thus promoting larval survival to the adult spawning stock, i.e. successful recruitment, is a major contributing factor to achieving this objective.

### Supplementary Information

Supplementary Information.

## Data Availability

All data associated with this study are available through the University of Western Australia’s research repository (https://research-repository.uwa.edu.au/en/datasets/).
