# Supplementary material for: Brief floodplain inundation provides growth and survival benefits to a young-of-year fish in an intermittent river threatened by water development
Source: Sci Rep. 2023 Oct 18;13:17725. doi: 10.1038/s41598-023-45000-x (PMC10584965; doi:10.1038/s41598-023-45000-x)
Supplement: Supplementary file 1 — Supplementary Information. [file 41598_2023_45000_MOESM1_ESM.pdf]

**<u>Supplementary material.</u>**

**Brief floodplain inundation provides growth and survival benefits to a young-of-year fish in an intermittent river threatened by water development**

Oliver P. Pratt*, Leah S. Beesley, Bradley J. Pusey, Daniel C. Gwinn, Chris S. Keogh and Michael M. Douglas

*Corresponding author: Oliver P. Pratt. Email: oliver.pratt@research.uwa.edu.au

Residual vs fitted values and Q-Q plots used for model fit diagnostics from the growth rate model fitted within a Bayesian framework.

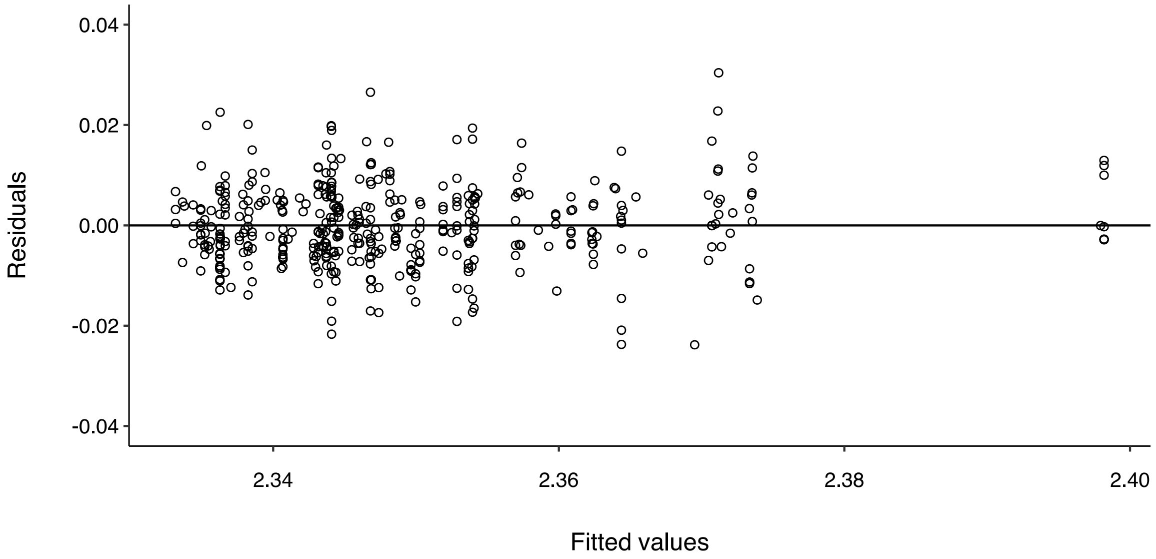


Figure S1 - Plot of residuals verses fitted values derived from growth rate linear model. An even spread of data points around the horizontal line indicate assumptions of homoscedasticity have been met.

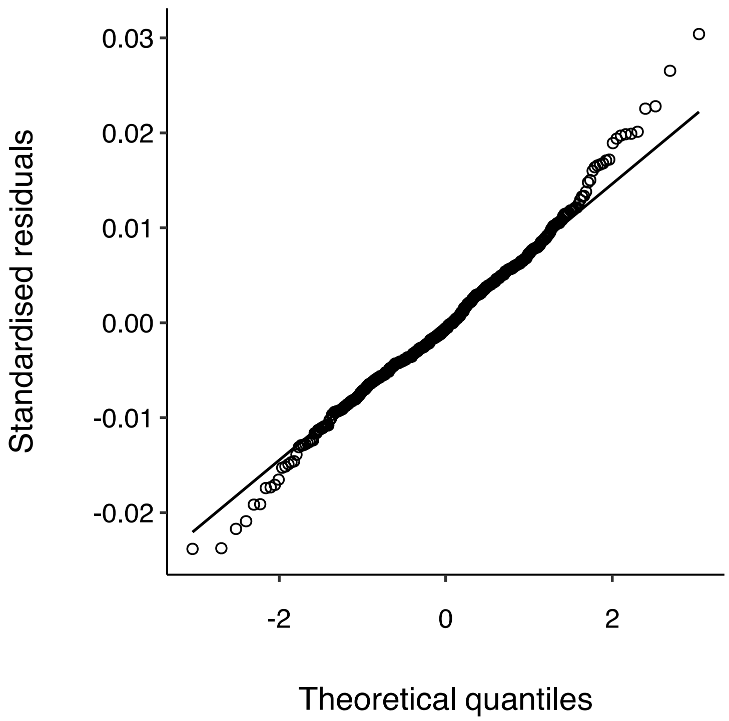


Figure S2- Plot of standard residuals verses theoretical quantiles derived from growth rate linear model. Data that plot approximately along the 1:1 line indicate assumptions of homoscedasticity have been met.
